# Nuts and Dried Fruits: An Update of Their Beneficial Effects on Type 2 Diabetes

**DOI:** 10.3390/nu9070673

**Published:** 2017-06-28

**Authors:** Pablo Hernández-Alonso, Lucía Camacho-Barcia, Mònica Bulló, Jordi Salas-Salvadó

**Affiliations:** 1 Human Nutrition Unit, Biochemistry and Biotechnology Department, Faculty of Medicine and Health Sciences, University Hospital of Sant Joan de Reus, IISPV, Universitat Rovira i Virgili, St/Sant Llorenç 21, 43201 Reus, Spain; pablo1280@gmail.com (P.H.-A.); marialucia.camacho@urv.cat (L.C.-B.); 2 CIBERobn Physiopathology of Obesity and Nutrition, Instituto de Salud Carlos III, 28029 Madrid, Spain

**Keywords:** diabetes, nuts, dried fruits, insulin resistance, mechanisms, clinical trials

## Abstract

Nuts and dried fruit are essential foods in the Mediterranean diet. Their frequent consumption has been associated with the prevention and/or the management of such metabolic conditions as type 2 diabetes (T2D), metabolic syndrome and cardiovascular diseases. Several previous reviews of epidemiological studies and clinical trials have evaluated the associations of nuts and/or dried fruit with various metabolic disorders. However, no reviews have focused on the mechanisms underlying the role of nuts and/or dried fruit in insulin resistance and T2D. This review aims to report nut and dried-fruit nutritional interventions in animals and humans, and to focus on mechanisms that could play a significant role in the prevention and treatment of insulin resistance and T2D.

## 1. Introduction

Nuts and traditional dried fruit (i.e., with no added sugar) are key food categories in the Mediterranean diet and other regional diets [1]. Several prospective studies, clinical trials and research in animals have reported beneficial effects after nut consumption [2]. However, the benefits of dried fruits (DF), mainly raisins, have been less explored [3].

Over time, food consumption has varied. More than 30 years ago, the consumption of nuts and DF was discouraged because of their high fat and sugar content, respectively. However, at the beginning of the 1990s, several randomized clinical trials (RCT) and animal experiments demonstrated their potential beneficial effect on cardiovascular diseases (CVD). Nuts and DF contain various macro and micronutrients together with other important bioactive compounds that may synergically contribute to modulate specific metabolic diseases such as hypercholesterolemia, hypertension and type 2 diabetes (T2D) (reviewed in [3,4]). Even so, the specific role of nuts and DF in the development and progression of insulin resistance (IR) and T2D are still controversial.

In this review, we focus on the role of nuts and DF in the prevention and treatment of T2D. We summarize published in vivo, in vitro, epidemiological and clinical studies, and we review the potential mechanisms that could explain the beneficial role of nut consumption on glucose and insulin metabolism, both of which are altered in T2D and in other glucose-impaired states. Given that the present article is not a systematic review, we may not have identified some studies and publication bias should be acknowledged. However, all authors independently conducted the literature search.

### 1.1. Nuts and Dried Fruits: The Concept

#### 1.1.1. Nuts

Nuts have been part of the human diet since prehistoric times [5,6]. They are an independent food group and are one of the cornerstones of the Mediterranean diet (MedDiet) [7]. According to the botanical definition, a nut is simply a dried fruit with one seed (rarely two) in which the ovary walls are very hard (stony or woody) at maturity, and the seed is unattached or free within the ovary wall. However, the word “nut” is commonly used to refer to any large, oily kernel in a shell that can be eaten as food. In this review, we use the term “nuts” to refer to almonds, Brazil nuts, cashews, hazelnuts, macadamias, peanuts, pecans, pine nuts, pistachios and walnuts. Although peanuts are actually classified as legumes because of their similar nutrient composition and their proven cardiovascular health benefits, they are commonly regarded as being a nut.

#### 1.1.2. Dried Fruits

To extend their shelf life, fresh fruits are processed by various techniques to become DFs [3]. Dried fruits are a concentrated form of fresh fruits with a lower moisture content. Fruits can be dried whole (e.g., apricots, berries and grapes), in halves, or in slices (e.g., kiwis, mangoes and papayas). In this form, they are easy to store and distribute, they can be available throughout the year, and they are a healthier alternative to salty or sugary snacks. Apples, apricots, currants, dates, figs, peaches, pears, prunes, and raisins are referred to as “conventional” or “traditional” DFs. Meanwhile, such fruits as blueberries, cranberries, cherries, strawberries and mangoes are commonly infused with different types of sugar solution (or fruit juice) concentrate before drying [8] so are not included in the aforementioned category. Moreover, we have also excluded dried tomato because although it is botanically a berry-type fruit, it is culinary considered a vegetable and it shares nutrient composition with this food category. 

### 1.2. Nutritional Composition of Nuts and Dried Fruits

Nuts and DFs are a matrix of important bioactive compounds such as Vitamins (Vitamin E, niacin, choline and/or folic acid), minerals (magnesium, potassium, calcium and/or phosphorus), phenolic compounds, carotenoids and/or phytosterols [9]. Importantly, some nuts and DFs are among the 50 foods with the highest antioxidant capacity [10] and are also a known source of bioactive compounds, including plant sterols [11]. Pistachios are particularly rich in β-carotenes which have been widely associated with a protective T2D role [12,13]. In addition, pistachios are the only nuts that contain significant amounts of lutein and zeaxanthin [9]. Sun-dried raisins retain the minerals and most of the phytochemicals and antioxidants of the grape, including its resveratrol [14,15]. In fact, sun-drying enhances the antioxidant content of raisins. Because of the dehydration process, phytonutrients are more concentrated in raisins than in grapes. However, the concentration of some compounds is decreased by the sun-drying process in DFs and by dry roasting techniques in nuts [9]. Polyphenols and tocopherols from nuts and DF have proved to be rapidly accessible in the stomach, thus maximizing the possibility of absorption in the upper small intestine, and contributing to the beneficial relation between nut and DF consumption and health-related outcomes [8,16].

However, their macronutrient compositions are quite different, which means that their energy contents are also quite different. Nuts contain a high amount of total fat (Range (Re): 43.9–78.8%) with a high amount of unsaturated fat (monounsaturated fatty acids (MUFA) + polyunsaturated fatty acids (PUFA), Re: 31.6–62.4%), a relatively low amount of carbohydrates (CHOs) (Re: 11.7–30.2%) and vegetable protein (Re: 7.9–25.8%) (Table 1).

Conversely, DFs are mainly composed of CHOs (Re: 61.3–72.8%). They have a low content of protein (Re: 0.17–4.08%) and a fat content of less than 1% (Table 2). Importantly, both foods also contain a considerable amount of dietary fiber. Overall, their unique and varied nutrient composition makes them key foods to counteract various metabolic diseases.

### 1.3. Diet Quality in the Context of Nut and Dried Fruit Consumption

Epidemiological studies conducted in children and adults have demonstrated a significant positive association between nut consumption and diet quality [17,18]. Furthermore, the results of a clinical trial conducted in obese (Ob) subjects (*n* = 124) showed that the nutritional dietary quality of nut consumers (reporting to eat 42 g hazelnuts/day for 12 weeks) was remarkably higher than among other groups consuming chocolate, potato crisps or no additional foods [19]. Moreover, including nuts in energy-restricted diets reduced attrition and increased weight loss, indicating that nuts enhance palatability and compliance with diets without compromising health [20].

Several studies have examined the associations of whole fruit or 100% fruit juice [21] with nutritional or health outcomes such as T2D but there is a lack of studies examining potential links between DF and diet quality. A prospective study conducted in adult participants (*n* = 13,292) in the 1999–2004 National Health and Nutrition Examination Survey (NHANES) demonstrated an association between DF consumption and diet quality [22]. DF consumption was associated with improved nutrient intakes, a higher overall diet quality score, and lower body weight (BW)/adiposity measures [22]. Moreover, in a cross-sectional study in healthy adults (*n* = 797) from Hong Kong, an inverse association has been found between the intake of vegetables, legumes, fruits, dried fruits and Vitamin C and the prevalence of metabolic disorders such as non-alcoholic fatty liver disease (NAFLD) [23].

The 2015 Dietary Guidelines for Americans included the following three healthy dietary patterns: a Healthy US-style Pattern, a Healthy Vegetarian Pattern and a Healthy Mediterranean-style pattern. Fruits, nuts, and seeds play a prominent role in all three of these food-based dietary patterns, which recommend 350–440 g/day of fruit, and 16–28 g/day of nuts and seeds [24].

## 2. In Vivo and In Vitro Studies

Even though much of the research on nuts, dried fruits and T2D is based on observational studies and human trials, some in vitro and in vivo studies also evaluate their modulatory effect on glucose and insulin metabolism. In this regard, the effect of nuts on glucose and insulin metabolism has been investigated by evaluating nut extracts [25,26] or nuts as a whole [27,28,29] mainly in mice or rats. However, dried fruit has been investigated—like their non-dried counterparts—mainly in in vitro studies [30,31,32,33]. Almost all the research has focused on the extracts from non-edible parts, such as the shell [34], leaves [35,36], stems [37] and roots [38], and very little on the nut or fruit kernel [27,28,29,39]. However, this review focuses on those studies evaluating specific nutrients (e.g., polyphenols) or edible parts in both traditional nuts and dried fruits with outcomes related to glucose metabolism, IR, and the T2D oxidation/inflammation axis [32,40].

### 2.1. Nuts

The in vivo studies on nuts and T2D-related parameters are summarized in Table 3. These studies were mainly performed using extracts from peanuts or walnuts. Peanut oil supplementation for 42 days in diabetes-induced rats significantly reduced glucose and glycated hemoglobin (HbA_1c_) concentrations and improved lipid metabolism compared to normal rats [27]. Other researchers have found similar improvements in glycaemia in genotypes of diabetes in rats fed with peanut oil extract [41,42] or peanut aqueous extract [25]. In the case of walnuts, a polyphenol-rich walnut extract (PWE) for 4 weeks significantly decreased urinary 8-hydroxy-2′-deoxyguanosin levels (an in vivo marker of oxidative stress) and improved serum TG in *db*/*db* mice [26]. Moreover, an HFD with a 21.5% of energy from walnuts tested in mice for 20 weeks significantly reduced TG compared to nut-free HFD and tended to improve glucose and IR [29].

In vitro assessment of the T2D-related antioxidant and inflammatory capacity of nuts has largely been conducted by examining the ability of extracts to increase the resistance of human plasma or low density lipoprotein (LDL) to oxidation. Extracts of walnut [43], almond and almond skins [44,45], pistachio [46], and hazelnut [47] have been found to increase the lag time oxidation of LDL. However, little research has focused on their in vitro effects on glucose and insulin metabolism. Specifically, a hydro-ethanolic extract of cashew nut and its principal compound, anacardic acid, significantly stimulated glucose uptake in C2C12 muscle cells in a concentration-dependent manner, suggesting that it may be a potential anti-diabetic nutraceutical [48]. Moreover, cytoprotective activity of pistachio extracts (methanolic, water or ethyl acetate) against oxidative (reactive oxygen species formation) and carbonyl stress has also been reported in a T2D model in hepatocytes from rats [49].

### 2.2. Dried Fruits

Both in vitro and in vivo research has mostly focused on grape. Overman and collaborators showed that a grape powder extract (GPE) significantly attenuated lipopolysaccharide (LPS)-mediated inflammation in macrophages and decreased the capacity of LPS-stimulated human macrophages to inflame adipocytes and cause IR [30]. Moreover, GPE further attenuated tumor necrosis factor-α (TNF-α) mediated inflammation and IR in primary cultures of human adipocytes [31]. Grape polyphenol extract modulated in vitro membrane phospholipid fatty acid (FA) composition but also decreased muscle TG content and increased muscle glucose transporter type 4 (GLUT4) expression in high-fat-high-sucrose diet-fed rats. Overall, it improved insulin resistance status (i.e., HOMA-IR parameter) [50]. This is of considerable importance because the accumulation of muscle TG content and the modification of the muscle phospholipid fatty acid pattern may have an impact on lipid metabolism and increase the risk of developing T2D [51]. Mice fed with grape skin extract showed hypoglycaemic and anti-hyperglycaemic effects (independent of an increase in insulin release) but are probably dependent on an increase in insulin sensitivity resulting from the activation of the insulin-signaling cascade in skeletal muscle [52]. Furthermore, grape seed aqueous extract protected the pancreas against oxidative stress, inflammation and apoptosis-induced damage while preserving pancreatic function at near normal levels in diabetic rats [53].

Other DF extracts have also been evaluated. Treatment with a date fruit extract was effective at decreasing behavioral, neurophysiological, and pathological alterations induced by diabetes in the peripheral nerves of streptozotocin (STZ)-induced diabetic rats (well-characterized animal model of type 1 diabetes) [33]. Moreover, daily consumption of a low- or high-fat diet supplemented with 1% black currant powder extract (with 32% of anthocyanins) for 8 weeks reduced body weight gain and improved glucose metabolism [54].

The beneficial effect of fruit juices and fermented grape juice (i.e., wine) have also been an important focus of research. Schmatz and collaborators investigated the ex vivo effects of a moderate consumption of red wine (RW) and grape juice (GJ), and the in vitro effects of various substances (resveratrol, caffeic acid, gallic acid, quercetin and rutin) on STZ-induced diabetic rats. They demonstrated decreased platelet aggregation in diabetic-induced rats after moderate RW and GJ consumption for 45 days [55]. Similarly, resveratrol increased the hydrolysis of adenosine triphosphate (ATP), while quercetin decreased it in platelets [55]. These results were extended using a wine grape powder supplementation, which prevented hyperglycemia and IR, and reduced oxidative stress in a rat model of metabolic syndrome (MetS) [56].

Specific compounds found in both nuts and DFs were also further investigated. Quercetin and trans-resveratrol are plant polyphenols which have showed a significant reduction of IR and inflammation associated with obesity. Eid et al. showed that quercetin (isolated from lingonberry) exerted an anti-diabetic activity by stimulating adenosine monophosphate-activated protein kinase (AMPK) [40]. Moreover, quercetin also enhanced basal glucose uptake in mouse myoblast C2C12 muscle cells in the absence of insulin [40] via a mechanism which is highly analogous to metformin. Importantly, quercetin seems to be as effective as or more effective than resveratrol in attenuating TNF-α-mediated inflammation and IR in primary human adipocytes and macrophages [32,57].

## 3. Epidemiological Studies on Nuts

The relationship between the consumption of different food categories and the incidence or prevalence of metabolic disorders has been explored all over the world. As is shown in Table 4, numerous epidemiological studies have assessed the associations between nut consumption and T2D. However, no studies have been published on the link between DF consumption and risk of T2D.

As far as nuts are concerned, several studies have found an inverse association between the frequency of consumption and the development of this metabolic pathology [58,59,60,61,62,63,64]. In the Nurses’ Health Study (NHS) and NHS II cohort, the intake of different types of nut was explored [61,64]. Total nut, walnut and peanut butter intake were associated with a lower risk of T2D in women. In the NHS (*n* = 83,818 female subjects), those with the highest nut consumption (28 g/day; ≥5 days a week) had a lower relative risk (RR) of developing T2D (0.73 [95% confidence interval (CI), 0.60–0.89]) than those who never/almost never consume (0.92 [95% CI, 0.85–1.00]) [61]. Results were similar for peanut butter: the RR was 0.79 (95% CI, 0.68–0.91) in those women who had a higher intake (5 times or more a week) than those who never/almost never ate peanut butter [61]. After combining NHS and NHS II cohorts, in a total of 137,953 female subjects, Pan and collaborators found that walnut consumption was inversely associated with risk of T2D [64].

These results are in line with those of a cross-sectional study of 7,210 subjects at high CV risk within the context of the PREvención con DIeta MEDiterránea (PREDIMED) study, where the upper category of nut consumption had a lower prevalence of T2D than the lower category [63]. A recent prospective study also associated the consumption of nuts—higher than 4 times a week—with a lower risk of T2D [60]. A cross-sectional study performed in the context of the National Health and Nutrition Examination Survey (NHANES) established a relation between the homeostatic model assessment of insulin resistance (HOMA-IR) and tree nut consumption. Decreased insulin resistance and lower levels of β-cell function markers were found in the nut consumers than the non-consumers [59]. In a large cohort of the Netherlands Cohort Study (NLCS), the total nut intake was associated to lower T2D cause-specific mortality in men and women [65].

Even though current evidence shows that nuts have a strong protective effect against the progress of T2D, especially in women, some epidemiological studies have not identified this relation [66,67]. The latest systematic review and meta-analysis designed to assess the relation between nut consumption and risk of cancer and T2D was published in 2015. It included five studies that were linked to T2D. After pooling data from studies conducted in both genders, it was found that there was no statistically significant association between nut consumption and risk of developing T2D (RR = 0.98 [95% CI, 0.84–1.14]), even though the heterogeneity was significant [68].

## 4. Human Clinical Trials

The effect of nut consumption on glucose and insulin metabolism has also been researched in acute and chronic clinical trials. Acute studies mostly demonstrate a decrease in postprandial glycaemia and hyperinsulinemia after nut consumption. In contrast, chronic randomized clinical trials designed to analyze the effects on glucose metabolism provided controversial results.

### 4.1. Nuts

#### 4.1.1. Acute Clinical Trials on Nuts

Table 5 summarizes various acute clinical studies of nut consumption, most of which focus on almonds [69,70,71,72,73]. In a randomized crossover trial in healthy subjects, almond intake decreased postprandial glycaemia and insulinaemia [69]. In a dose-response study conducted in healthy individuals, almond intake attenuated the postprandial glycaemic response of white bread [70]. In impaired glucose-tolerant subjects, it was observed that the consumption of almonds with a meal also decreased blood glucose in plasma [71]. In a recent crossover study performed in pre-diabetic subjects, a preload of almonds decreased postprandial glycaemia [73]. In healthy and diabetic individuals, Cohen et al. also showed a 30% reduction in postprandial glycaemia after almond consumption compared with a starchy meal [72].

The effect of pistachio intake on postprandial glycaemia was investigated by Kendall et al. in two randomized studies. In overweight (Ow) healthy subjects, pistachio consumption was reported to have a minimal effect on postprandial glycaemia. When pistachios were included in a carbohydrate meal, the relative glycaemic response (RGR) was attenuated [74]. In a crossover trial with 20 subjects with MetS, postprandial glycaemia decreased after the consumption of pistachios (85 g) compared to white bread. In the same study, a peripheral increase in the glucagon-like peptide-1 (GLP-1) concentrations was also observed after pistachio consumption compared with the consumption of white bread [75].

For peanuts, a randomized crossover trial conducted in 13 healthy subjects reported a decreased postprandial glycaemic response after the consumption of a breakfast containing 63 g of one of the following types of peanuts: raw with skin, roasted without skin and ground-roasted without skin [76]. Similarly, in a parallel study conducted in 65 overweight and obese men, peanut consumption reduced postprandial insulinaemia levels compared to high-oleic peanut consumption [77].

Nut consumption was observed to have similar beneficial effects on glucose and insulin metabolism in the only study to analyze the effect of a mix of nuts (almonds, macadamias, walnuts, pistachios, hazelnuts and pecans). In this study conducted in 10 diabetic and 14 non-diabetic subjects, nut consumption decreased the RGR compared to white bread. Importantly, this study also reported that nut consumption improved short-term glycaemic control in patients with T2D [74].

In summary, nuts seem to have beneficial postprandial glycaemic effects when consumed alone or in combination with high carbohydrate foods, and so may potentially help to prevent and manage impaired glucose states.

#### 4.1.2. Chronic Clinical Trials on Nuts

Most of the RCTs have compared nut-enriched diets with control diets in order to analyze their effects on lipid profile, and blood glucose and insulin concentrations as a secondary outcome. However, some, mainly conducted in subjects with T2D [72,79,80,81,82,83,84], but also in pre-diabetic [85], hyperlipemic [86], overweight/obese [87] and healthy individuals [88], were specifically designed to assess changes in glucose or insulin metabolism after nut consumption (Table 6).

In 2002, Lovejoy and coworkers evaluated the effect of an almond-enriched diet in 20 healthy and 30 T2D subjects in two different studies [79]. Healthy subjects, supplemented with 100 g almonds/day for 4 weeks and advised to reduce their energy intake by an equivalent amount, did not change their insulin sensitivity, whereas their body weight increased and their lipid profile improved. In a second study, subjects with T2D were randomized following a crossover design to one of 4 diets with different fat contents (25% or 37%) with 10% of fat from almonds or from olive or canola oil, with a minimum washout of 2 weeks between periods. Fat source (almond vs. oil) or fat level (high fat vs. low fat) were not observed to have any significant effect on either the glucose or insulin index. In contrast, in a crossover study conducted in 20 Chinese patients with T2D and mild-hyperlipidemia assigned to either a control diet (National Cholesterol Education Program (NCEP) step II diet) or an almond-enriched diet (with almonds replacing the 20% total daily calorie intake) for 4 weeks, a significant decrease in fasting insulin and glucose concentrations together with an improvement of HOMA-IR were reported during the almond phase [80]. In a similar crossover study conducted in 48 diabetic patients who received 50 g/day of pistachios and a pistachio-free diet (12 weeks each period), with an 8-week washout, a significant improvement in fasting blood glucose (FBG) and HbA_1c_ was observed during the pistachio consumption, while no changes in HOMA-IR were reported [83]. Recently, Gulati and coworkers conducted a pre-post intervention study in a group of 50 Asian Indians who consumed 20% of total energy in the form of whole raw almonds for 24 weeks preceded by a control diet free of nuts. Although no changes in FBG were observed during the almond consumption, the authors found a significant reduction in glycosylated hemoglobin, together with an improvement in other T2D risk factors such as waist circumference or inflammation status [84]. To determine whether the beneficial effect of nut consumption on glucose and insulin metabolism could also be extended to pre-diabetic subjects our group conducted a randomized crossover study in 54 pre-diabetic subjects who consumed a pistachio-supplemented diet (55 g pistachio/day) and a control diet (nut-free diet), each for 4 months with a 2-week washout period. We found a beneficial effect of pistachio intake on fasting glucose, insulin, and HOMA-IR. Other cardiometabolic risk markers such as fibrinogen, oxidized LDL, platelet factor 4 and GLP-1 were also modified appropriately during the consumption of pistachios [85].

Changes in fasting glucose or insulin levels, HOMA-IR and glycosylated hemoglobin have also been assessed as secondary outcomes in several clinical feeding trials with different designs (parallel, crossover) and subject characteristics (i.e., healthy, overweight/obese, TD2, MetS), mainly using walnuts and almonds, and with different intervention lengths (from 2 weeks to 2 years). The results obtained are controversial. Although most studies found no improvement in glucose/insulin metabolism [89,90,91,92,93,94,95,96,97,98,99,100,101,102,103], others reported a significant reduction in FBG levels [89,92,104,105], fasting insulin or insulin resistance [90,92,106,107] and HbA_1c_ [92]. However, a meta-analysis of RCTs including 25 trials with a total of 1,650 particpants who were otherwise healthy or had dyslipidaemia, metabolic syndrome or type 2 diabetes mellitus showed that the consumption of tree nuts led to modest decreases in fasting blood glucose compared with control diet interventions [108].

### 4.2. Dried Fruits

#### 4.2.1. Acute Clinical Trials on Dried Fruits

Less research has been carried out into DFs than into nuts. However, the findings to date point to a beneficial effect of DFs on postprandial glucose regulation and glycaemic control in T2D subjects.

The putative effect of DFs on postprandial glycaemia and insulinaemia has been studied mainly using raisins (Table 7). However, some research into dried plums has also been published.

First, in 1989 Rasmussen and coworkers evaluated in healthy and T2D subjects the postprandial effects of three meals: raw rolled oats, oatmeal porridge, or a mixture of raw rolled oats with raisins, compared to a control glucose ingestion [109]. The substitution of 25% of the starch meal with raisins (i.e., simple sugars) did not affect blood glucose or insulin responses. In addition, a similar glucose and insulin response in both normal and T2D subjects were reported [109]. Other researchers investigated the postprandial effect of raisins consumed alone. When the GI was investigated in three different groups (sedentary, aerobically trained or pre-diabetic subjects), no significant differences were found among groups, even though the GI (55–69) it seemed moderate for aerobically trained adults, and low (GI, ≤55) for the other groups [110]. Kanellos and collaborators found a moderate GI of raisins in healthy and T2D subjects [111], whereas Esfahani et al. found that raisins were low-GI and glycaemic load (GL) foods in healthy subjects [112]. Recently, researchers have found that even though the same available CHO content from raisins and glucose generated a similar postprandial response, raisins significantly modulated the levels of GIP, ghrelin and ghrelin/obestatin ratio, with important implications in terms of appetite regulation and overall insulin secretion [113]. In overweight women, researchers determined that dried plums had a lower plasma glucose and insulin incremental area under the curve (IAUC) than an isoenergetic low-fat cookie meal [114].

Overall, results suggest that raisins have a beneficial postprandial glucose and insulin effect, which may cautiously be extrapolated to other DFs considering their overall macronutrient composition.

#### 4.2.2. Chronic Clinical Trials on Dried Fruits

The study of the beneficial effects of chronic DF consumption also focuses on raisins (Table 8). Randomized clinical trials have been conducted in healthy [115], overweight or obese [116,117], T2D subjects [118,119], or a combination of the three [120]. In a 6-week parallel trial in healthy subjects, Puglisi et al. included 150 g/day of raisins into subjects’ habitual diet or increased their physical activity (or combined them both). Neither FPG nor insulin levels were different among groups or compared to baseline. However, the inflammation status reported by plasma TNF-α significantly decreased in the raisin intervention [115]. 

The results for overweight or obese subjects do not show a significant improvement in glucose or insulin levels after the consumption of raisins [116] or dried plums [117]. However, these studies are only short (2 weeks) which makes it impossible to analyze the chronic effect. In fact, in a parallel study conducted in Ow/Ob subjects, comparing raisin consumption (270 Kcal/day) with a snack (300 Kcal/day) for 12 weeks, researchers [120] found that even though FGP or insulin were not affected by either intervention, HbA_1c_ levels and postprandial glucose levels had been reduced by raisin consumption by the end of the trial. This suggests a beneficial effect of raisin consumption on glycaemic control in Ow/Ob subjects with pre-D. Importantly, raisin intake also improved systolic blood pressure (SBP) and diastolic blood pressure (DBP) and had a null effect on body weight [120].

Two studies have been conducted in individuals with T2D. A parallel study comparing the consumption of 36 g of raisins versus the habitual diet free of raisins or grapes for 24 weeks did not find any change in either body weight or in glycaemic control and lipid profile, but the total antioxidant capacity increased and the DBP decreased after raisin consumption [118]. Likewise, the consumption of raisins as a snack (84 g/day, 270 Kcal/day) for 12 weeks significantly reduced the postprandial glucose response in T2D subjects compared to an alternative snack (300 Kcal/day) for 12 weeks. A non-significant trend to a reduction in fasting glucose and HbA_1c_ was also observed in the same group of raisin consumers [119].

The results of DF consumption on glycaemia/insulinaemia point to a beneficial effect. However, novel acute and long-term RCTs assessing other types of DF should be carried out in order to corroborate and expand what is known about raisins.

## 5. Potential Mechanisms Linking Nut and Dried Fruit Consumption to Glucose and Insulin Metabolism

### 5.1. Nut- and Dried Fruit-Related Nutrients and Their Role in Glucose and Insulin Metabolism

#### Fiber Content in Nuts and Dried Fruits

Both nuts and DFs are high in dietary fiber [9]. Diets rich in complex CHO and fiber are associated with increased insulin sensitivity and reduced plasma insulin levels, promoting better glycaemic control in diabetic patients [121]. Soluble fiber increases gastric distension, viscosity in the gastrointestinal tract, and slower absorption of macronutrients [122]. As a consequence, the speed of CHO absorption and the concentration of postprandial glucose tend to be lower after the ingestion of fiber-rich foods than foods or meals poor in fibers [123].

Fiber is resistant to enzymatic digestion in the small intestine and thus susceptible to fermentation by bacteria in the colon. It produces short chain fatty acids (SCFA, e.g., acetate, propionate and butyrate) which reduce the production of hepatic glucose and stimulate the secretion of GLP-1 [124,125]. Incretins such as GLP-1 and gastric inhibitory polypeptide (GIP) stimulate the secretion of insulin by β-cells and promote the proliferation of these cells, favoring the maintenance of normal blood glucose levels [125]. The secretion of GLP-1—which is mainly performed by enteroendocrine L-cells of the gastrointestinal tract -is partly mediated by monosaccharides, peptides and amino-acids, MUFA and PUFA as well as by SCFA. Therefore, the positive influence of GLP-1 on blood glucose homeostasis, appetite sensations, and food intake provides a strong rationale for its therapeutic potential in the nutritional management of T2D and obesity [126].

Overall fiber contained in nuts and DFs is also able to decrease postprandial glycaemic levels and this could be a strategy for increasing insulin sensitivity which improves T2D and several other CV risk factors for chronic diseases [74,127].

#### Carbohydrate Content—Glycaemic Index of Nuts and Dried Fruits

It should be noted that nuts are relatively low in CHO (approximately 15% of the total energy) whereas DFs have a high amount of CHO (60–80%). Nuts have a low glycaemic index and therefore increase the blood glucose level less and require less insulin secretion, thus favoring the control of T2D. However, because DFs are high in carbohydrates and fiber, their specific GI has been the object of considerable study. The GI of raisins was first evaluated in three heterogeneous groups of subjects (aerobically trained, sedentary or pre-diabetic) and was described between 49 and 69, therefore corresponding to the low-to-moderate GI foods [110]. However, later studies have reported that raisins are in the low GI category in healthy subjects (a GI of 49.4 and an insulinemic index of 47.1) [15]. This suggests a favorable postprandial glucose and insulin response [112], that could be explained by the high proportion of fructose that DFs contain.

Overall, the inclusion of both nuts and DFs in a balanced diet may reduce the overall glycaemic index of the diet, with benefits to glycaemic and insulinemic control in both healthy and T2D subjects [128].

#### Fat Content in Nuts

The high content of MUFA and PUFA in nuts seems to enhance the reduction of IR, thus consequently reducing the risk of developing T2D [129,130,131]. However, the mechanisms by which these fatty acids (FA) affect insulin sensitivity are not yet fully understood [132]. It is believed, however, that the FAs present in the phospholipids of different cell membranes are affected by the type of fatty acid intake, thus affecting insulin sensitivity. The different unsaturated FAs that are part of the cell membrane influence the action of insulin via affecting the binding or affinity of insulin to its cellular receptor [133]. It is hypothesized that a higher unsaturation of FA in the cell membrane facilitates the movement of the glucose receptor to the cell surface, thus increasing insulin sensitivity [134]. In addition, as we have seen, unsaturated fatty acids act through the stimulation of GLP-1 secretion, thus improving the efficiency of β-cells. SFA and MUFA act on the lipogenic gene expression, while PUFA inhibit the expression of these genes, partly by binding and activating nuclear receptors, such as those activated by peroxisome proliferators (PPAR) [131]. It has been suggested that the activation of PPAR has a therapeutic role in the treatment of T2D, because it induces fatty acid clearance by the adipose tissue, decreasing its plasma levels and thus increasing insulin sensitivity in the muscle [135].

#### Mineral Content in Nuts and Dried Fruits

Nuts contain relatively high quantities of different minerals such as potassium, magnesium and calcium, whereas DF contains more moderate quantities (mainly potassium, magnesium and boron). Magnesium has been associated with beneficial effects on glycaemic control [136]. Low magnesium levels have been implicated in IR development [124]. In fact, kidneys lose the ability to retain magnesium in periods of acute hyperglycemia. It is excreted in the urine, leading to low mineral blood levels. It has been shown that correcting this depletion improves response and insulin action. Magnesium deficiency also interferes with the reactions that use or produce ATP, thus altering the enzyme cascade involved in the CHO metabolism and favoring the development of T2D [137].

Therefore, the minerals present in nuts and DF could explain the relationship observed between magnesium intake and the lower risk of developing T2D and other chronic diseases [138,139].

#### Other Bioactive Compounds in Nuts and Dried Fruits

The other bioactive compounds contained in nuts and DF could partially explain their protective anti-oxidant and anti-inflammatory properties [2] and their implication in glucose and insulin metabolism (and therefore in T2D). Significant evidence suggests that polyphenol-rich diets have the ability to protect against diabetes [140]. It appears that anthocyanins (or anthocyanin-rich foods) are inversely associated to the risk of T2D, but there is no association for other polyphenol subclasses [141,142]. Dietary polyphenols have some benefits for T2D: they protect pancreatic β-cells against glucose toxicity and they have anti-inflammatory and anti-oxidant effects, among other things. Quercetin is a member of the flavonoid class of polyphenols which is abundant in such foods as fruits, vegetables, nuts and seeds [143]. Several in vitro studies have sought to elucidate the mechanisms behind the antidiabetic properties of quercetin: for example the inhibition of the α-amylase and α-glucosidase activities, and the prevention of the lipid peroxidation of pancreatic tissue homogenates [144]. Moreover, ellagic acid (EA) is another polyphenol which naturally occurs in some fruits, such as berries (strawberries and red and black raspberries), nuts, and pomegranates [145]. There is emerging in vitro evidence that EA may ameliorate symptoms of chronic metabolic diseases—by decreasing chronic inflammation at the expression level—which include dyslipidemia, IR and T2D [146,147,148]. Despite the growing amount of information on EA, a definitive mechanism of action has not been established. This may be attributed to the complexity of EA metabolism, which is governed by various factors [145].

A growing body of evidence suggests that dietary agents and the non-nutrient components of fruits, vegetables and nuts can affect epigenetic processes related to T2D [149,150]. Epigenetics generally refers to heritable and reversible changes affecting gene expression and chromatin organization that are not due to alterations in the DNA sequence [151,152]. No specific data is available on the role of nuts and DFs in T2D epigenetics. However, dietary polyphenol resveratrol—most abundant in the skin of grapes and raisins, but also found in peanuts and cranberries [153]—and other phytochemicals (e.g., curcumin) have proved to be effective agents against cancer and to act through epigenetic mechanisms that affect the global epigenome [154,155]. However, future studies are needed to determine the biological importance of the altered tissue-specific DNA methylation in T2D resulting from nutritional modifications [152].

### 5.2. Cellular and Molecular Mechanisms Linking Nut and Dried Fruit Consumption and the Prevention and/or Management of T2D/IR

#### Gene Expression

Some studies have analyzed the effect of nut and DF consumption on changes in the gene expression of particular cells and/or tissues and its relationship with glucose and insulin metabolism or T2D. In particular, a crossover study conducted in pre-diabetic subjects consuming pistachio for 4 months reported a downregulation of interleukin-6 (*IL-6*) and resistin (*RETN*) genes in leukocytes, whereas the pistachio diet led to a lower significant increase of solute carrier family 2 member 4 (*SLC2A4*, codifying GLUT4) [85]. Consistent with this attenuation in the expression of glucose transporters, leukocytes significantly reduced the uptake of glucose following pistachio consumption, suggesting a decrease in the cell hyperactivity described in T2D.

To our knowledge no published studies have evaluated gene expression after the consumption of DF. However, a translational study conducted in 120 healthy young subjects has shown that individuals in the highest tertile of fruit and vegetable consumption had statistically lower values of the expression of some pro-inflammatory marker genes such as intercellular adhesion molecule-1 (ICAM-1), IL-6 and TNF-α, in peripheral blood mononuclear cells (PBMC) [156].

Therefore, these results shed new light on the nutrient-gene interactions in nuts and DFs. However, further research is needed.

#### MicroRNAs

The modulatory role of nuts and DF on the expression of genes related to inflammation, oxidative stress and glucose metabolism can also be mediated by the effect of nutrients on microRNAs. MicroRNAs (miRNAs) are RNA molecules that belong to a family of small non-coding RNAs with 20 to 25 nucleotides [157,158] that post-transcriptionally and negatively regulate gene expression.

Research into the nutritional modulation of miRNA is still in its infancy and few studies have assessed whether a specific dietary pattern, food, supplements or a particular nutrient can influence miRNA levels [159]. In humans, consumption of grape extract rich in resveratrol, and Vitamin supplementation modulated specific miRNAs towards a healthier status [160]. Similarly, a PUFA-enriched diet including almonds and walnuts was effective at modifying several miRNAs [161]. More recently, pistachio consumption in pre-diabetic subjects significantly diminished the levels of miR-375 and miR-192, both related to the glucose and insulin metabolism [162]. However, there is still much to discover about the exact mechanisms linking miRNAs with the glucose and insulin metabolism and the dietary modulation of miRNA expression.

#### Microbiota and Metabolomic Modulation

In recent years, many studies have pointed out that the gut microbiome might make an important contribution to the development of insulin resistance and T2D. Several mechanisms related to the composition of gut microbes—including changes in bowel permeability, endotoxemia and interaction with bile acids—may contribute to the onset of insulin resistance. On the other hand, it is well established that long-term dietary patterns and shorter-term dietary variation influence gut microbiota composition [163]. Many potential prebiotic components can be present in a particular food. For example, the fermentation of fiber from nuts and DF to beneficial end-products (e.g., butyric acid) and the biotransformation of phytochemicals (i.e., tocopherols, phytosterols, and phenolic compounds) are associated with the transition to a healthier microbiota composition [2,164].

There is little information on the putative effect of nuts and DF on microbiota modulation. In fact, only two publications have evaluated the effect of nut consumption (almonds and pistachios) on changes in microbiota. Mandalari et al. conducted an in vitro study with almond skins and they found that almond fiber significantly altered the composition of gut bacteria, specifically *Bifidobacteria*, thus showing that almond skins had a potential prebiotic use [165]. Some years later, Ukhanova and collaborators carried out two separate crossover feeding trials with nuts and pistachios, and showed that they both significantly affected microbiota modulation [166]. However, a greater prebiotic effect was found after pistachio intake. In fact, pistachio increased the number of butyrate-producing bacteria, which are potentially beneficial, whereas the numbers of *Bifidobacteria* were not affected by the consumption of either nut [166].

Recently, dietary polyphenols have been found to be involved in gut microbiota dysbiosis processes which may lead to a reduction of fat storage, inflammation and IR (reviewed in [167]). In fact, a cranberry extract also altered the gut microbiome of mice by increasing mucin-degrading bacteria, a potential link to reverse the dysbiosis and metabolic inflammation underlying T2D [168].

Very few studies have been made on metabolomic modulation after nut and DF intake. Of the studies that have been made, some have evaluated changes in metabolites after a chronic consumption of nuts. They showed a modulation in the levels of metabolites such as raffinose, (12Z)-9,10-dihydroxyoctadec-12-enoate, sucrose, together with some modulations of plasma amino acids and fatty acids [169], and a modulation of gut-related metabolites and cis-aconitate, an intermediate of tricarboxylic acid [170], after pistachio consumption.

**Figure 1:**
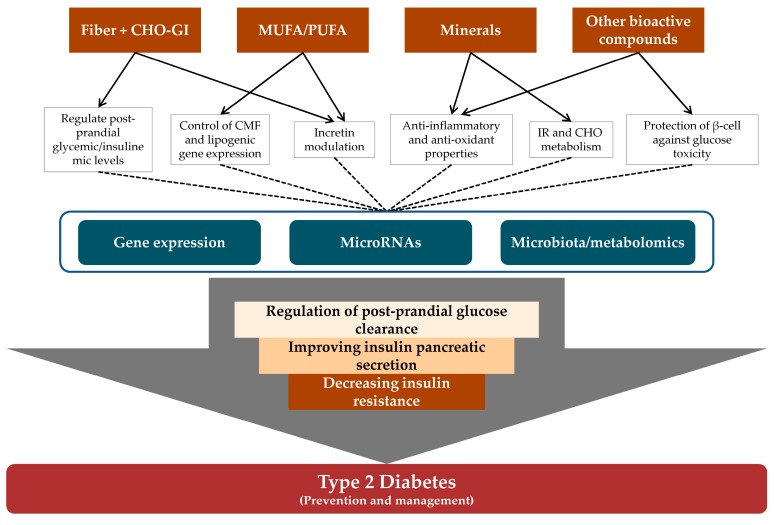
Role of nutrients from nuts and dried fruits in glucose and insulin metabolism, and cellular and molecular mechanisms related to T2D/IR. CHO, carbohydrate; CMF, cellular membrane fluidity; GI, glycaemic index; IR, insulin resistance; MUFA, monounsaturated fatty acid; PUFA, polyunsaturated fatty acid; T2D, type 2 diabetes.

Therefore, several specific nutrients—together with their synergic effects—in both nuts and DFs may explain their beneficial role in glucose and insulin metabolism, which helps to prevent or maintain T2D (Figure 1).

## 6. Concluding Remarks

Undoubtedly, the specific composition of nuts and dried fruits means that they can be used to efficiently counteract metabolic diseases such as type 2 diabetes. Their unique profile of macronutrients, micronutrients and other bioactive compounds may explain the beneficial effects observed in clinical and epidemiological studies. However, the exact mechanisms by which they modulate glucose and insulin metabolism and influence T2D have yet to be fully discovered. They contain fiber, fat, minerals and other bioactive molecules that modulate several gene mechanisms at the cellular and molecular level. This may explain some of their beneficial effects. However, further basic and translational research is needed in order to extend their positive health benefits and to find novel mechanisms and targets to explain their contribution to the management of type 2 diabetes.

## Figures and Tables

**Table 1:** Nutrient composition of nuts (per 100 g of raw nut).

Nutrient	Almonds	Brazil Nuts ^b^	Cashews	Hazelnuts	Macadamias	Peanuts	Pecans	Pine Nuts ^b^	Pistachios	Walnuts ^c^
Energy, Kcal	579	659	553	628	718	567	691	673	560	654
Water, g	4.4	3.4	5.2	5.3	1.4	6.5	3.5	2.3	4.4	4.1
Fat, g	49.9	67.1	43.9	60.8	75.8	49.2	72.0	68.4	45.3	65.2
SFA, g	3.8	16.1	7.8	4.5	12.1	6.3	6.2	4.9	5.9	6.1
MUFA, g	31.6	23.9	23.8	45.7	58.9	24.4	40.8	18.8	23.3	9.0
PUFA, g	12.3	24.4	7.8	7.9	1.5	15.6	21.6	34.1	14.4	47.2
Protein, g	21.2	14.3	18.2	15.0	7.9	25.8	9.2	13.7	20.2	15.2
CHO, g	21.6	11.7	30.2	16.7	13.8	16.1	13.9	13.1	27.2	13.7
Fiber, g	12.5	7.5	3.3	9.7	8.6	8.5	9.6	3.7	10.6	6.7
Ca, mg	269	160	37	114	85	92	70	16	105	98
Mg, mg	270	376	292	163	130	168	121	251	121	158
Na, mg	1	3	12	0	5	18	0	2	1	2
K, mg	733	659	660	680	368	705	410	597	1025	441
P, mg	481	725	593	290	188	376	277	575	490	346
Lutein-Zeaxanthin, µg	1	0	22	92	NA	0	17	9	2903	9
β-Carotene, µg	1	0	0	11	NA	0	29	17	305	12
α-Carotene, µg	0	0	0	3	NA	0	0	0	10	0
Phytosterols ^a^, mg	197	123.5	151	122	116	NA	158.8	236.1	214	110.2
Total phenols, mg	287	244	137	687	126	406	1284	32	867	1576
Vitamin E (α-tocopherol), mg	25.6	5.7	0.9	15.0	0.5	8.3	1.4	9.3	2.9	0.7

Nutrient information is taken from the United States Department of Agriculture (USDA) Nutrient Database Standard Reference, Release 28 [9]. CHO, carbohydrates; MUFA, monounsaturated fatty acids; NA: not available; PUFA, polyunsaturated fatty acids; SFA, saturated fatty acids. ^a^ Phytosterols, are the sum of stigmasterol, campesterol, β-sitosterol and other phytosterols; ^b^ dry roasted; ^c^ English variety.

**Table 2:** Nutrient composition of dried fruits (per 100 g).

Nutrient	Apples ^a^	Apricots ^a^	Currants (Zante)	Cranberries ^b^	Dates ^c^	Figs	Peaches ^a^	Pears ^a^	Plums/Prunes	Raisins ^d^
Energy, Kcal	243	241	283	308	282	249	239	262	240	299
Water, g	31.76	30.89	19.21	15.79	20.53	30.05	31.80	26.69	30.92	15.43
Fat, g	0.32	0.51	0.27	1.09	0.39	0.93	0.76	0.63	0.38	0.46
CHO, g	65.89	62.64	74.08	82.80	75.03	63.87	61.33	69.70	63.88	79.18
Sugars, g	57.19	53.44	67.28	72.56	63.35	47.92	41.74	62.20	38.13	59.19
Fructose, g	NA	12.47	NA	26.96	19.56	22.93	13.49	NA	12.45	29.68
Protein, g	0.93	3.39	4.08	0.17	2.45	3.30	3.61	1.87	2.18	3.07
Fiber, g	8.7	7.3	6.8	5.3	8.0	9.8	8.2	7.5	7.1	3.7
Ca, mg	14	55	86	9	39	162	28	34	43	50
Fe, mg	1.40	2.66	3.26	0.39	1.02	2.03	4.06	2.10	0.93	1.88
Mg, mg	16	32	41	4	43	68	42	33	41	32
Na, mg	87	10	8	5	2	10	7	6	2	11
K, mg	450	1162	892	49	656	680	996	533	732	749
Cu, mg	0.19	0.34	0.47	0.06	0.21	0.29	0.36	0.37	0.28	0.32
β-carotene, µg	0	2163	43	27	6	6	1074	2	394	0
α-carotene, µg	0	0	1	0	0	0	3	0	57	0
Lutein-Zeaxanthin, µg	0	0	0	138	75	32	559	50	148	0
Vitamin A, IU	0	3604	73	46	10	10	2163	3	781	0
Total phenols, mg GAE/100g ^e^	324	248	NA	NA	661	960	283	679	938	1065

Data is for traditional dried fruits which is defined as those with no added sugars, typically sun-dried or dried with minimal processing. Nutrient information is taken from the United States Department of Agriculture (USDA) Nutrient Database Standard Reference, Release 28 [9]. CHO, carbohydrates; GAE, gallic acid equivalents; IU, international unit; NA, not available. ^a^ Sulfured; ^b^ sweetened; ^c^ Deglet noor is the common variety; ^d^ seedless; ^e^ Total phenol content was obtained from Alasalvar and Shahidi [8].

**Table 3:** Summary of in vivo studies and their characteristics in the context of nut consumption and type 2 diabetes (T2D)-related outcomes.

First Author (Year) [Reference]	Nut (Study Length)	Animal Model Used	Control	Intervention	Glucose and Insulin Metabolism Effects	Other Outcomes
Bilbis, L.S.; et al. (2002) [25]	Aqueous extract of peanut (21 days)	Alloxan-induced diabetic rats (*n* = 12) and non-diabetic rats (*n* = 12), divided into 3 equal groups	Non-diabetic with unrestricted standard diet and: (a) water ad libitum; (b) unrestricted access to drinking water and 2 mL of the extract 3 times/day; or (c) free access to the extract as the only drinking water.	Diabetic controls: treated as (a), (b) or (c)	The extract (alone or plus water) decreased FBG in both normal and alloxan-induced diabetic rats.	Significant decrease in serum TG, TC, HDL-C and LDL-C in both normal and alloxan-induced diabetic rats.
Fukuda, T.; et al. (2004) [26]	Polyphenol-rich walnut extract (PWE) (4 weeks)	*db/db* (*n* = 15) and C57BL/KsJ-*db*/*db* (*n* = 6) mice	Control *db*/*db* mice (*n* = 8) and C57BL/KsJ-*db*/+ m mice (*n* = 6, used for the blank group) were given water.	Experimental *db*/*db* mice (*n* = 7) received oral PWE (200 mg/kg BW)	Significant decrease in the level of urinary 8-hydroxy-2′-deoxyguanosin (in vivo marker of oxidative stress) in PWE-fed mice	Serum TG level was improved after PWE administration
Ramesh, B.; et al. (2006) [27]	Peanut oil (42 days)	Normal (*n* = 12) and STZ-diabetes induced (*n* = 18) Wistar rats	G1: Normal rats G3: Diabetic rats	G2: Normal rats + peanut oil diet (2%) G4: Diabetic rats + peanut oil diet (2%) G5: Diabetic rats + GLI (600 µg/kg BW)	Diabetic rats fed with peanut oil significantly reduce glucose, HbA_1C_, and G6Pase and FBP activities	Diabetic rats fed with peanut oil showed a small but significant reduction in TC, VLDL-C, LDL-C and TG and an increase in HDL-C.
Vassiliou, E.K.; et al. (2009) [41]	Peanut oil (21 days)	Male KKA^y^ (*n* = 24) mice	KKA ^y^ mice fed with normal diet (11.4% fat)	Diabetic KKA^y^ + HFD. Diabetic KKA^y^ + HFD with peanut oil (0.70 mL/day). HFD is 58% fat.	Diabetic mice administered peanut oil had lower glucose levels than animals administered HFD alone.	
Choi, Y.; et al. (2016) [29]	Walnuts (20 weeks)	Male C57BL/6J mice (≥6 mice/group)	Regular rodent chow	HFD (45% energy-derived) with or without walnuts (21.5% energy-derived)	Glucose and insulin resistance tended to improve with walnut supplementation.	Walnut supplementation did not change the HFD-induced increase in BW or VFM. However, dietary walnuts significantly decreased the amounts of hepatic TG observed in HFD-fed mice.
Adewale, O.F.; et al. (2016) [42]	Peanut oil Palm oil (3 weeks)	Normal (*n* = 12) and alloxan-induced diabetic Wistar rats (*n* = 36)	Non-diabetic	Diabetic non-supplemented. Diabetic supplemented with PeO or PaO (200 mg/kg/day)	Significant reduction in blood glucose of supplemented groups (PeO + PaO) compared to the diabetic non-supplemented group.	Plasma Vitamins C and E and albumin levels were significantly increased in the supplemented groups versus the diabetic non-supplemented group.

BW, body weight; FBG, fasting blood glucose; FBP, fructose-1,6-bisphosphatase; G6Pase, glucose 6-phosphatase; GLI, glibenclamide; HDL-C, high-density lipoprotein cholesterol; HFD, high-fat diet; LDL-C, low-density lipoprotein cholesterol; PaO, palm oil; PeO, peanut oil; PWE, polyphenol-rich walnut extract; STZ, streptozotocin; T2D, type 2 diabetes; TC, total cholesterol; TF, tissue factor; TG, triglycerides; VFM, visceral fat mass; VLDL-C, very low-density lipoprotein.

**Table 4:** Summary of epidemiological studies evaluating nut consumption.

First Author (Year) [Reference]	Study Name (Design)	Number of Subjects	Years of Follow-Up	Exposure	Findings
Jiang, R.; et al. (2002) [61]	NHS (Prospective)	83,818 women	16	≥5 times/week vs. never/almost never	Nut and peanut butter consumption was inversely associated with the risk of incident T2D.
Nettleton, J.A.; et al. (2008) [58]	MESA (Prospective)	5011 men and women	5	Quintiles of low-risk food pattern	High intake of whole grains, fruit, nuts/seeds, and green leafy vegetables was inversely associated to the risk of incident T2D.
Villegas, R.; et al. (2008) [62]	SWHS (Prospective)	64,227 women	4.6	Quintiles of peanut consumption	Consumption of peanuts was associated with a decreased risk of incident T2D.
Kochar, J.; et al. (2010) [67]	PHS I (Prospective)	20,224 men	19.2	≥7 servings of nuts/week vs. rarely or never consumers	No statistically significant association was found between nut consumption and T2D in either lean or overweight/obese subjects.
Ibarrola-Jurado, N.; et al. (2013) [63]	PREDIMED (Cross-sectional)	7210 at high cardiovascular risk	Baseline	<1 serving/week, 1–3 servings/week and >3 servings/week	The upper category of nut consumption had a lower prevalence of T2D than the lowest category.
Pan, A.; et al. (2013) [64]	NHS, NHS II (Prospective)	137,953 women	10	1–3 servings/month, 1 serving/week, and ≥2 servings/week of walnuts vs. never/rarely	Higher walnut consumption is associated with a significantly lower risk of T2D incidence.
O’Neil, C.E.; et al. (2015) [59]	NHANES (Cross-sectional)	14,386 men and women	6	Tree nut consumption compared with no consumption	Tree nut consumption was associated with lower HOMA-IR
Buijsse, B.; et al. (2015) [66]	EPIC-InterAct Study (Case-cohort)	16,154 men and women	12.3 Incident cases of T2D at 6.8	Non-consumers vs. the middle tertile of consumption.	Consumption of nuts and seeds does not modify T2D risk under isocaloric conditions and independent from BMI.
Asghari, G.; et al. (2017) [60]	TLGS (prospective)	1984 men and women	6.2 ± 0.7	≥4 servings/week vs. 1 or <1 serving/week	Nut consumption was associated with a lower risk of T2D incidence.

BMI, body mass index; CVD, cardiovascular disease; HOMA-IR, homeostatic model assessment of insulin resistance; MI, myocardial infarction; T2D, type 2 diabetes. Study name acronyms: EPIC, European Prospective Investigation into Cancer; HPFS, Health Professionals Follow-Up Study; MESA, Multi-Ethnic Study of Atherosclerosis; NHANES, National Health and Nutrition Examination Survey; NHS, Nurses’ Health Study; NLCS, Netherlands Cohort Study; PHS, Physicians' Health Study; PREDIMED, PREvención con DIeta MEDiterránea; SCCS, Southern Community Cohort Study; SMHS, Shanghai Men's Health Study; SWHS, Shanghai Women's Health Study; TLGS, Tehran Lipid and Glucose Study.

**Table 5:** Summary of acute clinical studies analyzing the effect of nut consumption on postprandial response.

First Author (Year) [Reference]	N° of Subjects (M/F) Type of Subject (Age in Years)	Type of Nut (Study Design)	Control Group	Intervention Group	Glucose and Insulin Metabolism Outcomes	Other Outcomes
Jenkins, D.J.; et al. (2006) [69]	15 (7/8) Healthy subjects (26.3 ± 8.6)	Almonds (crossover)	97 g of white bread	- Almond meal: 60 g almonds +97 g bread - Parboiled rice meal: 68 g cheese and 14 g butter +60 g parboiled rice - Mashed potato meal: 62 g cheese and 16 g butter +68 g mashed potatoes	Almonds decrease postprandial glycaemia and insulinaemia.	Almonds are likely to decrease oxidative damage to serum proteins by decreasing glycaemic excursion and providing antioxidants.
Josse, A.R.; et al. (2007) [70]	9 (7/2) Healthy subjects (27.8±6.9)	Almonds (crossover dose-response study)	White bread	- White bread +30 g almonds - White bread +60 g almonds - White bread +90 g almonds	The 90-g almond meal resulted in a significantly lower GI than the white bread control meal	
Mori, A.M.; et al. (2011) [71]	14 (8/6) IGT (39.3 ± 10.9)	Almonds (crossover)	75 g of available CHO (No almonds)	75 g of available CHO from: - Whole almonds - Almond butter - Defatted almond flour - Almond oil	Whole almonds significantly attenuated second-meal and daylong blood glucose IAUC.	GLP-1 concentrations did not significantly vary between treatments.
Kendall, C.W.; et al. (2011) [74]	10 (3/7) Ow healthy subjects (48.3 ± 6.4)	Pistachios (crossover)	White bread	Study 1: - 28, 56 and 84 g pistachios - 28, 56 and 84 g Study 2: - 56 g of pistachios + different commonly consumed carbohydrate foods (50 g available carbohydrate).	Pistachios consumed alone had a minimal effect on postprandial glycaemia. Pistachios consumed with a carbohydrate meal attenuated the RGR.	
Cohen, A.E. and Johnston, C.S. (2011) [72]	20 (6/14) Healthy subjects (*n* = 13) and T2D subjects (*n* = 7) (Healthy: 53.0 ± 3 and T2D: 66.0 ± 3.3)	Almonds (postprandial: crossover trial)	No almond meal	28 g almonds enriched meal	The ingestion of almonds immediately before a starchy meal significantly reduced postprandial glycaemia by 30%.	
Kendall, C.W.; et al. (2011) [78]	24 (11/13) Healthy (*n* = 14) and T2D subjects (*n* = 10) (Healthy: 36.0 ± 4 and T2D: 68.0 ± 2)	Mixed nuts (i.e., almonds, macadamias, walnuts, pistachios, hazelnuts and pecans) (crossover)	White bread	3 doses of 30, 60 and 90 g of mixed nuts	Nuts improve short-term glycaemic control in patients with T2D.	
Reis, C.E.; et al. (2011) [76]	13 (4/9) Healthy subjects (28.5 ± 10)	Peanuts (crossover)	Cheese sandwich	63 g of: - raw peanuts with skin - roasted peanuts without skin - ground-roasted peanuts without skin	The ingestion of ground-roasted peanuts without skin for breakfast leads to a lower CHO intake and reduced postprandial glycaemic response.	
Moreira, A.P.; et al. (2014) [77]	65 men Ow/Ob (Range: 18–50)	Conventional peanuts and high-oleic peanuts (parallel)	56 g biscuit	- 56 g conventional peanuts (*n* = 21) - 56 g high-oleic peanuts (*n* = 23)	Conventional peanut consumption was associated with decreased postprandial insulinaemia, which might be beneficial for saving β-cell function, independently of the influence on LPS concentrations.	
Kendall, C.W.; et al. (2014) [75]	20 (8/12) Subjects with MetS (54.0 ± 8)	Pistachios (crossover)	Control 1: white bread Control 2: (white bread + butter + cheese)	Test meal 1: WB + 85 g of pistachios Test meal 2: 85 g of pistachios	Pistachio consumption reduced postprandial glycaemia compared with white bread.	Pistachio consumption increased GLP-1 levels compared with white bread.
Crouch, M.A. and Slater, R.T. (2016) [73]	20 (13/7) Subjects with pre-diabetes * (Mean: 60.8)	Almonds (crossover)	No almonds	12 units of dry-roasted almonds	A low-calorie almond preload “appetizer” decreased postprandial hyperglycemia.	

Age is shown as mean ± SD unless otherwise stated. BMI, body mass index; CHO, carbohydrate; GLP-1, glucagon-like peptide-1; HbA_1c_, glycated hemoglobin; IAUC, incremental area under the curve; IGT, impaired glucose tolerance; LPS, lipopolysaccharide; MetS, metabolic syndrome; M/F, male/female; Ob, obese; Ow, overweight; RGR, relative glycaemic responses; T2D, type 2 diabetes; WB, white bread. * also include “isolated 1-h glucose > 160 mg/dL”.

**Table 6:** Summary of chronic clinical trials and their characteristics in the context of nut consumption.

First Author (Year) [Reference]	N° of Subjects (M/F) Type of Subjects (Age in Years)	Nut Study Design (Length of the Intervention)	Control Group	Intervention Group(s)	Glucose and Insulin Metabolism Outcomes	Other Outcomes
Lovejoy, J.C.; et al. (2002) [79]	30 (13/17) T2D subjects (mean ± SEM: 53.8 ± 1.9)	Almonds Crossover (1 month per period)	HF-Control LF-Control	HF-HA LF-HA	No significant changes in glycaemia were observed.	Total cholesterol was lowest after the HF-HA diet. HDL-C was significantly decreased after the almond diet; however, no significant effect of fat source on LDL: HDL was reported.
Jenkins, D.J.A.; et al. (2008) [86]	27 (15/12) Hyperlipidemic subjects (64 ± 9)	Almonds Crossover (1 month per period)	147 ± 6 g/day of muffins	Almonds (73 ± 3 g/day) Half portion of almonds (37 ± 2 g/day) plus muffins (75 ± 3 g/day) Isoenergetic (mean, 423 Kcal/day)	No significant changes were observed in FBG, insulin, C-peptide, or HOMA-IR. The 24-h urinary C-peptide output, as a marker of 24-h insulin secretion, was significantly reduced by the half-and full-dose almonds in comparison to the control muffin diet after adjustment for urinary creatinine output.	There were no significant treatment differences in BW.
Claesson, A.L.; et al. 2009 [88]	25 (11/14) Healthy subjects (range: 19–30)	Peanuts Parallel (2 weeks)	Addition of 20 kcal/kg-BW of candy to the regular caloric intake.	Addition of 20 kcal/kg-BW of roasted peanuts to the regular caloric intake.	Plasma-insulin and C-peptide increased in the candy group, but not in the peanut group. FBG was not modified.	Energy intake increased similarly in both groups. BW and WC increased significantly only in the candy group. At the end of the study LDL-C and ApoB/ApoA-1-ratio were higher in the candy group than in the peanut group.
Cohen, A.E.; et al. (2011) [72]	13 (7/6) T2D subjects (66.0 ± 3.3)	Almonds Parallel (3 months)	Nut- free diet	Diet enriched with almonds (28 g, 5 times/week)	Significant reduction of HbA_1c_ in the almond group compared to the nut-free diet group.	Chronic almond ingestion was associated with a reduction in BMI as compared with no change in the nut–free diet group.
Li, S.C.; et al. (2011) [80]	20 (9/11) T2D subjects (Mean: 58)	Almonds Crossover (1 month per period)	NCEP step II diet (control diet); CHO (56 E%), protein (17 E%), and fat (27 E%).	Almonds were added to the control diet to replace 20% of total daily calorie intake.	Compared with subjects in the control diet, those in the almond diet reduced the levels of fasting insulin, FBG, and HOMA-IR.	Almond intake decreased TC, LDL-C, and LDL-C/HDL-C. The almond diet enhanced plasma α-tocopherol level compared with control diet.
Damavandi, R.D.; et al. (2013) [81]	45 (15/33) Medicated T2D subjects (55.68 ± 7.74)	Hazelnuts Parallel (2 months)	Control diet	10% of total daily calorie intake was replaced with hazelnuts	No significant differences in FBG between groups.	No changes in BMI were reported. Significant HDL-C reduction in control group was observed. Although the hazelnut group achieved a greater reduction in TG concentrations than the control group, these changes were non-significant.
Hernández-Alonso, P.; et al. (2014) [85]	54 (29/25) Subjects with Pre-D Mean: 55 (range: 53.4–56.8)	Crossover (4 months per period)	Nut-free diet: the energy intake of other fatty foods, mostly olive oil, was adjusted to compensate for the energy from pistachios included in the PD.	Pistachio diet was supplemented with 2 ounces of pistachio (57 g/day)	FBG, insulin, and HOMA-IR decreased significantly after the chronic pistachio period compared with the nut-free period.	Fibrinogen, oxidized-LDL, and PF-4 significantly decreased under the pistachio period compared to the nut-free period, whereas GLP-1 increased.
Lasa, A.; et al. (2014) [82]	191 (77/114) T2D subjects (Mean: 67)	Mixed nuts Parallel (1 year)	LFD	Mediterranean diets supplemented with either virgin olive oil or mixed nuts	Increased values of the adiponectin/leptin ratio and adiponectin/HOMA-IR ratio and decreased values of WC were observed in the three groups.	In both Mediterranean diet groups, but not in the LFD group, this was associated with a significant reduction in BW.
Parham, M.; et al. (2014) [83]	44 (11/33) T2D subjects (Mean: 51)	Pistachios Crossover (3 months per period)	Previous diet without pistachios	Two snacks of 25 g pistachios/day	Marked decrease in HbA_1c_ and FBG concentrations in the pistachio diet group compared with the control group.	There were no overall significant changes in BMI, blood pressure, HOMA-IR, or CRP concentrations.
Le, T.; et al. (2016) [87]	213 women Ow/Ob subjects (Mean: 50)	Walnuts Parallel (1 year)	Control 1: a lower fat (20 E%), higher CHO (65 E%) diet. Control 2: lower CHO (45 E%), higher fat (35 E%) diet	Walnut-enriched diet: high fat (35 E%), lower CHO (45 E%) diet.	Insulin sensitivity and CRP levels improved after walnut-rich diet	TG decreased in all study arms at 6 months. The walnut-rich diet increased HDL-C more than either the lower fat or lower CHO diet. The walnut-rich diet also reduced LDL-C.

Age is shown as mean ± SD unless otherwise stated. Apo, apolipoprotein; BMI, Body mass index; BW, body weight; CHO, carbohydrate; CRP, C-reactive protein; E%, energy percentage; FBG, fasting blood glucose; GLP-1, glucagon-like peptide-1; HA: high almond; HbA_1c_, glycated hemoglobin; HDL-C, high-density lipoprotein cholesterol; HF, high fat; HOMA-IR, homeostatic model assessment of insulin resistance; LDL-C, low-density lipoprotein cholesterol; LF: low fat; LFD, LF diet; M/F, male/female; NCEP, National Cholesterol Education Program; NS, non-significant; Ob, obese; Ow, overweight; PF-4, platelet factor-4; PM, post-menopausal; Pre-D, pre-diabetes; T2D, type 2 diabetes; TC, total cholesterol; TG, triglycerides; WC, waist circumference; WHtR, waist-to-height ratio.

**Table 7:** Summary of acute clinical studies analyzing the effect of dried fruit consumption on postprandial response.

First Author (Year) [Reference]	N° of Subjects (M/F) Type of Subject (Age in Years)	Dried Fruit (Study Design)	Control Group	Intervention Group(s)	Glucose and Insulin Metabolism Outcomes	Other Outcomes
Rasmussen, O.; et al. (1989) [109]	20 (9/11) Healthy (*n* = 11) and T2D subjects (*n* = 9) (Healthy: 30 ± 2; T2D subjects: 67 ± 2)	Raisins (crossover)	75 g (healthy) or 50 g (T2D) of CHO	Raw rolled oats; oatmeal porridge or a mixture of raw rolled oats with raisins	Substitution of 25% of the starch meal with raisins (simple sugars) did not affect blood glucose or insulin responses	In normal and T2D subjects, the three meals produce similar glucose and insulin response curves.
Kim, Y.; et al. (2008) [110]	10 S; 11 AT and 10 Pre-D (S (25.7 ± 1.3), AT (23.1 ± 1.0), Pre-D (50.0 ± 2.6))	Raisins (crossover)	50 g of available CHO from glucose	50 g of available CHO from raisins	NS differences among groups. The GI of raisins seemed lower (≤55) in the S and P groups compared to moderate (GI, 56–69) in the A group. The insulinaemic index of raisins was not different among groups.	
Furchner-Evanson, A.; et al. (2010) [114]	19 women ow subjects (39.2 ± 0.7)	Dried plums (crossover)	White bread (238 Kcal)	Dried plums (238 Kcal) Low-fat cookies (238 Kcal)	Dried plums elicited lower plasma glucose and insulin IAUC than low-fat cookies.	The satiety index IAUC was greater for the dried plums than low-fat cookies, and tended to promote a greater plasma ghrelin AOC
Kanellos, P.T.; et al. (2013) [111]	30 (17/13) Healthy and T2D subjects (*n* = 15 each) (Healthy: 25.9 ± 0.8; T2D: 63.2 ± 1.7)	Corinthian raisins (crossover)	50 g of glucose	74 g of Corinthian raisins; 50 g of available CHO	Significantly different glucose peaks between raisins and glucose in healthy and in diabetic subjects. Glycaemic and insulinaemic responses were decreased after raisin consumption compared to glucose ingestion.	
Esfahani, A.; et al. (2014) [112]	10 (4/6) Healthy subjects (39 ± 11)	Raisins (crossover)	108 g of white bread; 50 g available CHO (consumed on two separate occasions)	R50: 69 g raisins; 50 g available CHO R20: 28 g raisins; 20 g available CHO	The raisin meals, R50 and R20, resulted in significantly reduced postprandial glucose and insulin responses compared with white bread	Raisins were determined to be low in GI, GL and insulinaemic index.
Kaliora, A.C.; et al. (2017) [113]	10 Healthy normo-weight subjects (26.3 ± 0.8)	Raisins (crossover)	50 g of glucose	74 g of raisins; 50 g of available CHO	At 60 min, glucose and insulin levels were maximum in both groups.	GIP was lower after raising intake compared to glucose intake at 60 and 120 min postprandially. Ghrelin was lower after raisin compared to glucose intake at 120 and at 180 min post-ingestion. No differences were reported for GLP-1, apelin or obestatin in either trial.

Age is shown as mean ± SD unless otherwise stated. AT, aerobically trained; AOC, area over the curve; CHO, carbohydrates; GI, glycaemic index; GL, glycaemic load; GLP-1, glucagon-like peptide-1; IAUC, incremental area under the curve; M/F, male/female; NS, non-significant; ow, overweight; Pre-D, pre-diabetic; S, sedentary; T2D, type 2 diabetes; WB, white bread.

**Table 8:** Summary of chronic clinical trials and their characteristics in the context of dried fruit consumption.

First Author (Year) [Reference]	N° of Subjects (M/F) Type of Subject (Age in Years)	Study Design (Length of the Intervention)	Control Group	Intervention Group(s)	Glucose and Insulin Metabolism Outcomes	Other Outcomes
Puglisi, M.J.; et al. (2008) [115]	34 (17/17 PM) Healthy (range: 50–70)	Raisin Parallel (6 weeks)	Walk (increase in the steps taken per day)	150 g/day of raisins. Walk + 150 g/day of raisins	Changes in FBG and insulin values did not differ among intervention groups or from baseline. Plasma TNF-α decreased in the raisin group but no differences were reported between groups.	Plasma TC and LDL-C decreased in all the intervention groups.
Rankin, J.W.; et al. (2008) [116]	17 (8/9) Ow (26.5 ± 7.6)	Raisin Crossover (2 weeks per period)	Jelly candy (264 Kcal/day)	90 g/day raisins (264 Kcal/day)	NS changes in FBG or markers of inflammation or endothelial dysfunction after the raisin intervention.	Fasting protein-free ORAC was modestly higher after the raisin intervention than the jelly candy intervention.
Howarth, L.; et al. (2010) [117]	26 women Ow/Ob (range: 25–54)	Dried plums Crossover (2 weeks per period)	Low-fat cookies (200 Kcal/day)	Dried plums (200 Kcal/day)	No changes were found in plasma glucose or insulin levels in any intervention.	Plasma TG concentration was unchanged by dried plum consumption and was higher after the consumption of low-fat cookies. Incorporation of dried plums or low-fat cookies into the diet did not alter energy intake or BW.
Anderson, J.W.; et al. (2014) [120]	46 (21/25) Ow/Ob with Pre-D or at T2D risk. (snack (mean: 61.1), raisins (mean: 60.3))	Raisins Parallel (12 weeks)	Snacks (300 Kcal/day)	84 g/day of raisins (270 Kcal/day)	Fasting HbA_1c_ levels were significantly reduced after raisin intake, whereas FBG and insulin levels were not significantly affected by the intake of raisins or snacks. Postprandial glucose levels were significantly reduced by raisin intake vs. snacks.	Raisin intake was associated with reductions in SBP and DBP. BW did not significantly change within or between groups.
Kanellos, P.T.; et al. (2014) [118]	48 (25/23) T2D (raisins (63.7 ± 6.3), control (63 ± 8.5))	Corinthian raisins Parallel (24 weeks)	Usual diet avoiding grapes and raisins	36 g/day of Corinthian raisins	BW, glycaemic control, and lipid profile were not changed in either arm of the intervention. Patients in the CR arm reduced their DBP and increased their total antioxidant potential compared with baseline values and the control group.	No change in CRP was observed. A significant difference in plasma circulating p-hydroxybenzoic acid was observed between groups at the end of the trial.
Bays, H.; et al. (2015) [119]	46 (19/27) T2D (mean: 58)	Dark raisins Parallel (12 weeks)	Snack group (300 Kcal/day)	84 g/day of dark raisins group (270 Kcal/day)	Compared to the snack group, those who consumed raisins reduced their postprandial glucose levels, and an NS trend to a reduction in fasting glucose and HbA_1c_. NS changes in BW, fasting insulin, HOMA-IR or lipid profile between intervention groups.	Compared to alternative processed snacks, those who consumed raisins had a significant reduction in SBP but not a significant reduction in DBP.

Age is shown as mean ± SD unless otherwise stated. BW, body weight; CRP, C-reactive protein; DBP, diastolic blood pressure; FBG, fasting blood glucose; HbA_1c_, glycated hemoglobin; HOMA-IR, homeostatic model assessment of insulin resistance; LDL-C, low-density lipoprotein cholesterol; M/F, male/female; NS, non-significant; Ob, obese; ORAC, oxygen radical absorbance capacity; Ow, overweight; pre-D, pre-diabetes; PM, postmenopausal women; SBP, systolic blood pressure; T2D, type 2 diabetes; TC, total cholesterol; TG, triglycerides; TNF-α, tumor necrosis factor-α.

## Abbreviations

AMPK	adenosine monophosphate-activated protein kinase
AT	aerobically trained
ATP	adenosine triphosphate
AOC	area over the curve
Apo	apolipoprotein
BMI	body mass index
BW	body weight
C	cholesterol
CHO	carbohydrate
CI	confidence interval
CMF	cellular membrane fluidity
CRP	C-reactive protein
CVD	cardiovascular disease
DF	dried fruit
EA	ellagic acid
EPIC	European Prospective Investigation into Cancer
FA	fatty acid
FBG	fasting blood glucose
FBP	fructose-1,6-bisphosphatase
G6Pase	glucose 6-phosphatase
GAE	gallic acid equivalents
GI	glycaemic index
GIP	gastric inhibitory polypeptide
GL	glycaemic load
GJ	grape juice
GLI	glibenclamide
GLP-1	glucagon-like peptide-1
GLUT	glucose transporter type
GPE	grape powder extract
HA	high almond
HbA_1c_	glycated hemoglobin
HDL	high-density lipoprotein
HF	high fat
HFD	high-fat diet
HOMA-IR	homeostatic model assessment of insulin resistance
HPFS	Health Professionals Follow-Up Study
IAUC	incremental area under the curve
ICAM-1	intercellular adhesion molecule-1
IGT	impaired glucose tolerance
IL-6	interleukin-6
IR	insulin resistance
LDL	low-density lipoprotein
LF	low-fat
LPS	lipopolysaccharide
MedDiet	Mediterranean diet
MESA	Multi-Ethnic Study of Atherosclerosis
MetS	metabolic syndrome
MI	myocardial infarction
miRNA and miR	microRNA
M/F	male/female
MUFA	monounsaturated fatty acids
NA	not available
NAFLD	non-alcoholic fatty liver disease
NCEP	National Cholesterol Education Program
NHANES	National Health and Nutrition Examination Survey
NHS	Nurses’ Health Study
NLCS	Netherlands Cohort Study
NS	non-significant
Ob	obese
ORAC	oxygen radical absorbance capacity
Ow	overweight
PBMC	peripheral blood mononuclear cell
PF-4	platelet factor-4
PHS	Physicians' Health Study
PM	post-menopausal
PPAR	peroxisome proliferator-activated receptors
pre-D	pre-diabetes
PREDIMED	PREvención con DIeta MEDiterránea
PUFA	polyunsaturated fatty acids
PWE	polyphenol-rich walnut extract
Re	range
RETN	resistin
RCT	randomized clinical trial
RGR	relative glycaemic responses
RR	relative risk
RW	red wine
ROS	reactive oxygen species
S	sedentary
SBP	systolic blood pressure
SCCS	Southern Community Cohort Study
SCFA	short chain fatty acids
SFA	saturated fatty acids
SLC2A4	solute carrier family 2 member 4
SMHS	Shanghai Men's Health Study
STZ	streptozotocin
SWHS	Shanghai Women's Health Study
T2D	type 2 diabetes
TC	total cholesterol
TF	tissue factor
TG	triglycerides
TLGS	Tehran Lipid and Glucose Study
TNF-α	tumor necrosis factor-α
USDA	United States Department of Agriculture
VFM	visceral fat mass
WB	white bread
WC	waist circumference
*WHtR*	*waist-to-height ratio*
